# A positive linear correlation between the triglyceride-glucose index and in-stent restenosis after percutaneous coronary intervention in patients with coronary heart disease

**DOI:** 10.3389/fcvm.2025.1544125

**Published:** 2025-07-29

**Authors:** Jin Mao, Zigen Fang, Shan Jiang, Zeyan Xia

**Affiliations:** Department of Emergency Medicine, Zhongda Hospital, School of Medicine, Southeast University, Nanjing, China

**Keywords:** triglyceride-glucose index, in-stent restenosis, coronary heart disease, percutaneous coronary intervention, biomarker

## Abstract

**Aims:**

The association between the triglyceride-glucose (TyG) index and in-stent restenosis (ISR) after percutaneous coronary intervention (PCI) in patients with coronary heart disease (CHD) remains inadequately explored. This study aimed to evaluate the relationship between TyG and ISR in patients with CHD following PCI.

**Methods:**

This retrospective study included 519 patients with CHD undergoing PCI. TyG, considered as the exposure variable, was divided into lower (≤9.21) and higher (>9.21) groups based on the optimal cutoff determined by receiver operator characteristic (ROC) analysis, with ISR as the outcome variable. Multivariable logistic regression, subgroup analysis, ROC analysis and restricted cubic spline (RCS) modeling were used to assess the association between TyG and ISR.

**Results:**

Patients with higher TyG had a significantly greater incidence of ISR compared to patients with lower TyG (*P* = 0.003). Patients with ISR had higher levels of TyG compared with patients without ISR (*P* = 0.006). In multivariable logistic regression analysis, after adjusting for confounding variables, a higher TyG index was significantly associated with an increased risk of ISR, both as a categorical and a continuous variable [Model 3, OR (95% CI), *P* value: 1.786 (1.134, 2.814), 0.012 and 1.408 (1.034, 1.917), 0.030, respectively]. The association remained significant in subgroups aged < 60 years, male, non-smokers, and those with hypertension (*P* < 0.05). Additionally, ROC analysis showed that TyG had modest predictive value for ISR (AUC = 0.571, *P* = 0.020), and its addition to the baseline model significantly improved the overall predictive performance (AUC = 0.643, *P* < 0.001). RCS analysis further confirmed a positive linear correlation between TyG and ISR (*P* = 0.042; *P* for nonlinearity = 0.808).

**Conclusion:**

A higher TyG index is significantly associated with an increased risk of ISR in CHD after PCI, highlighting its potential as a valuable biomarker for cardiovascular risk stratification.

## 1 Introduction

In-stent restenosis (ISR) is defined as a narrowing of ≥50% in the arterial lumen within or immediately adjacent to a previously implanted stent, as determined by angiography, and is primarily the result of endothelial injury, neointimal hyperplasia, and vascular smooth muscle cell proliferation following percutaneous coronary intervention (PCI) (1, 2). Although the reported incidence of ISR varies across studies due to differences in study populations and methodologies, it has historically reached up to 55% in the pre-stent century. Even with bare-metal stents, ISR rates remained as high as 41%. Encouragingly, with advances in interventional techniques and the widespread use of drug-eluting stents, the incidence of ISR has declined to below 18% (3, 4). Nevertheless, ISR continues to be associated with poor cardiovascular outcomes in patients with coronary heart disease (CHD), highlighting the urgent need to identify and manage modifiable risk factors early in the clinical course (4). Although Giustino et al. conducted a systematic review demonstrating that procedural, anatomic, and stent-related factors are closely linked to ISR development, additional metabolic or systemic risk factors may also play a role (4). A recent systematic review by Wilson et al. showed that diabetes was strongly associated with the development of ISR (5). Notably, diabetes is also a well-established risk factor for cardiovascular disease, chronic kidney disease, and adverse events (6–8). Importantly, the harmful vascular effects associated with diabetes may already be present in its early stages, or even during the preceding phase of insulin resistance (IR) (9, 10).

IR, a marker of systemic inflammation and metabolic dysfunction, is distinct from both diabetes and prediabetes (11). It is difficult to assess in routine clinical settings and often receives inadequate attention. Although the euglycaemic-hyperinsulinaemic clamp (EHC) remains the gold standard for IR evaluation, it is rarely used in practice due to its complexity, high cost, and limited reproducibility (12). Therefore, numerous simplified surrogate markers of IR have been developed. Among them, triglyceride-glucose (TyG) index has emerged as one of the most reliable. Derived from a simple formula combining fasting triglyceride (TG) and fasting blood glucose (FBG) levels, the TyG index has been proved to be strongly related to EHC. Compared to other IR surrogate indicators such as homeostasis model assessment index (HOMA-IR), TyG has several advantages: it does not require insulin measurements, is easier to calculate, more stable, and based on routinely available laboratory parameters, which makes it highly applicable in large-scale clinical and epidemiological studies (13). Therefore, TyG is increasingly used as a key IR marker in metabolic research (13). At present, numerous studies have confirmed the correlation between TyG and cardiovascular disease and its poor prognosis (14, 15). However, limited data exist regarding the relationship between TyG and ISR in patients with CHD undergoing PCI. Therefore, this retrospective cohort study aimed to investigate the association between TyG and ISR in patients with CHD after PCI.

## 2 Subjects, materials and methods

### 2.1 Study population

This study was a secondary analysis based on a cohort of hospitalized patients with CHD, details of which have been described previously (16, 17). After excluding individuals who lacked key data necessary to determine ISR status, including follow-up coronary angiography results, fasting TG or FBG, a total of 519 patients were included in the analysis. The study protocol complied with the Declaration of Helsinki and was approved by the Ethics Committee of the Zhongda Hospital, Affiliated to Southeast University. Informed consent was waived for all patients due to the retrospective nature of the study.

### 2.2 Data collection and definitions

All the data in this study were obtained from a publicly available dataset (17), including demographic data, comorbidities, medication history, biomarkers data, echocardiographic parameters, coronary angiographic data, and follow-up data. The following variables were included for analysis: age, sex, smoking status, diabetes, hypertension, stroke, previous PCI, use of aspirin, clopidogrel, β-blockers, angiotensin-converting enzyme inhibitors (ACEIs), calcium channel blockers (CCBs), statins, left ventricular ejection fraction (LVEF), body mass index (BMI), systolic blood pressure (SBP), diastolic blood pressure (DBP), TG, total cholesterol (TC), low-density lipoprotein cholesterol (LDL-C), high-density lipoprotein cholesterol (HDL-C), estimated glomerular filtration rate (eGFR), uric acid, FBG, number of diseased vessels, left main disease, multi-vessel disease, restenotic lesions, number of treated vessels, number of stents, total stent length, stent type, and ISR status.

Smoking was defined as a history of smoking within the past 10 years. BMI was calculated as weight (kg) divided by height squared (m^2^). TyG was defined as Ln [fasting TG (mg/dl) × FBG (mg/dl)/2] (18). The optimal cut-off point for predicting ISR was determined by receiver operating characteristic (ROC) curve analysis. Based on the The Youden index, which maximizes the sum of sensitivity and specificity, was used to identify the optimal threshold of 9.21. Patients were then categorized into two groups: lower TyG group (TyG ≤ 9.21) and higher TyG group (TyG > 9.21). Diabetes was defined as FBG ≥ 7.0 mmol/L, glycosylated hemoglobin ≥ 6.5% or current use of hypoglycemic drugs (19). Hypertension was defined as SBP/DBP ≥ 140/90 mmHg or use of antihypertensive drugs (20). The eGFR was calculated using a modified version of the Modification of Diet in Renal Disease (MDRD) equation consistent with the Chinese population (21).

### 2.3 Assessment of ISR

All patients underwent at least one repeat coronary angiogram at a median follow-up time of 29.8 months after PCI. Angiographic evaluations were performed by two independent, experienced cardiologists blinded to the study protocol, with discrepancies resolved by a third senior cardiologist. ISR was defined as a significant stenosis (≥50%) of the arterial lumen within or immediately adjacent to the stent, as confirmed by angiography (1). In this study, all patients were divided into two groups: non-ISR group and ISR group.

### 2.4 Statistical analysis

The categorical variables were described by frequencies (percentages) and compared using the chi-square test or Fisher's exact test, as appropriate. For continuous variables, normality was assessed prior to analysis. If normally distributed, data were reported as mean ± standard deviation and compared using the independent samples t-test. If not, they were reported as median (interquartile range) and analyzed using the Mann–Whitney U test. Univariate logistic regression was first used to screen variables with *P* < 0.05 or clinical relevance. These variables were then included in three multivariable logistic regression models to evaluate the correlation between TyG and ISR. Model 1 adjusted for age and sex; model 2 adjusted for age, sex, smoking, diabetes, hypertension, stroke and previous PCI; and model 3 adjusted for variables in Model 2 plus clopidogrel, eGFR, HDL-C, restenosis lesion, number of treated vessels, number of stents and stent type. Then, patients were stratified into 10 subgroups according to age, sex, smoking status, diabetes, and hypertension to perform subgroup analysis. Results were presented using a forest plot. ROC analysis was used to evaluate the discriminatory ability of TyG for ISR, and the area under the curve (AUC) of the baseline model (including age, sex, smoking, diabetes, hypertension, stroke, previous PCI, clopidogrel, eGFR, HDL-C, restenosis lesion, number of treated vessels, number of stents, and stent type) with TyG was statistically compared to assess whether TyG provided significant incremental predictive value. Restricted cubic spline (RCS) analysis was used to assess the potential nonlinear association between TyG and ISR. The SPSS 27.0, MedCalc 19.6.1 and R 4.3.1 were used for statistical tests. A two-tailed *P* value < 0.05 was considered statistically significant.

## 3 Results

### 3.1 Baseline characteristics

As shown in Table 1, patients with higher TyG levels had younger age, higher prevalence of diabetes, hypertension and ISR, as well as higher levels of BMI, TG, TC, LDL-C, eGFR, uric acid, and FBG, and lower levels of HDL-C compared to those with lower TyG (*P* < 0.05).

**Table 1:** Baseline characteristics by the triglyceride-glucose index.

Variables	Total population	Lower TyG	Higher TyG	*P* value
*N*	519	367	152	
Age, years	58.90 ± 11.15	59.99 ± 11.29	56.27 ± 10.36	0.001
Sex, male, *n* (%)	345 (66.50%)	249 (67.80%)	96 (63.20%)	0.458
Smoking, *n* (%)	179 (34.50%)	128 (34.90%)	51 (33.60%)	0.773
Comorbidities, *n* (%)				
Diabetes	101 (19.50%)	50 (13.60%)	51 (33.60%)	<0.001
Hypertension	256 (49.30%)	168 (45.80%)	88 (57.90%)	0.012
Stroke	32 (6.20%)	24 (6.50%)	8 (5.30%)	0.582
Previous PCI	51 (9.80%)	38 (10.40%)	13 (8.60%)	0.530
Medication, *n* (%)				
Aspirin	517 (99.60%)	367 (100.00%)	150 (98.70%)	0.085
Clopidogrel	506 (97.50%)	358 (97.50%)	148 (97.40%)	0.578
β-blockers	375 (72.30%)	266 (72.50%)	109 (71.70%)	0.859
ACEIs	259 (49.90%)	183 (49.90%)	76 (50.00%)	0.977
Calcium channel blockers	119 (22.90%)	77 (21.00%)	42 (27.60%)	0.101
Statins	492 (94.80%)	350 (95.40%)	142 (93.40%)	0.363
LVEF, %	61.59 ± 6.42	61.30 ± 6.49	62.34 ± 6.19	0.177
Body mass index, kg/m^2^	23.66 ± 3.81	23.26 ± 3.90	24.51 ± 3.48	0.027
Systolic blood pressure, mmHg	109.85 ± 28.20	109.53 ± 27.90	110.65 ± 28.99	0.681
Diastolic blood pressure, mmHg	77.82 ± 12.09	77.24 ± 12.11	79.25 ± 11.95	0.087
Triglycerides, mmol/L	1.58 (1.08, 2.42)	1.26 (0.99, 1.72)	2.94 (2.36, 3.86)	<0.001
Total cholesterol, mmol/L	4.31 ± 1.08	4.12 ± 1.02	4.76 ± 1.08	<0.001
LDL-C, mmol/L	2.67 ± 0.95	2.58 ± 0.93	2.87 ± 0.97	0.002
HDL-C, mmol/L	1.06 ± 0.30	1.10 ± 0.31	0.96 ± 0.27	<0.001
eGFR, ml/min/1.73 m^2^	116.68 ± 43.63	113.72 ± 43.25	123.82 ± 43.87	0.017
Uric acid, umol/L	303.00 ± 99.82	296.79 ± 103.79	318.00 ± 88.02	0.029
Fasting blood glucose, mmol/L	5.15 (4.63, 6.24)	4.93 (4.49, 5.58)	6.30 (5.10, 8.52)	<0.001
Angiography				
Number of diseased vessels	1.13 ± 1.16	1.12 ± 1.15	1.15 ± 1.21	0.761
Left main disease, *n* (%)	24 (4.60%)	19 (5.20%)	5 (3.30%)	0.351
Multi-vessel disease, *n* (%)	129 (24.90%)	89 (24.30%)	40 (26.30%)	0.620
Restenotic lesions, *n* (%)	12 (2.30%)	8 (2.20%)	4 (2.60%)	0.753
Number of treated vessels	1.61 ± 0.71	1.59 ± 0.71	1.65 ± 0.71	0.380
Number of stents	2.37 ± 1.42	2.40 ± 1.46	2.32 ± 1.33	0.551
Total stent length, mm	55.11 ± 35.40	55.85 ± 36.42	53.33 ± 32.86	0.463
Stent type, *n* (%)				0.081
SES	319 (61.60%)	230 (62.80%)	89 (58.60%)	
PES	93 (18.00%)	57 (15.60%)	36 (23.70%)	
SES/PES	106 (20.50%)	79 (21.60%)	27 (17.80%)	
In-stent restenosis, *n* (%)	122 (23.50%)	73 (19.90%)	49 (32.20%)	0.003

Lower TyG: TyG ≤ 9.21, Higher TyG: TyG > 9.21. TyG, triglyceride-glucose index; PCI, percutaneous coronary intervention; ACEIs, angiotensin-converting enzyme inhibitors; LVEF, left ventricular ejection fraction; LDL-C, low-density lipoprotein cholesterol; HDL-C, high-density lipoprotein cholesterol; eGFR, estimated glomerular filtration rate; SES, sirolimus-eluting stent; PES, paclitaxel-eluting stent.

### 3.2 Association of TyG with ISR

As shown in Table 2, in the multivariable logistic regression model, a higher TyG index was significantly associated with an increased risk of ISR after adjusting for age, sex, smoking, diabetes, hypertension, stroke, previous PCI, clopidogrel, eGFR, HDL-C, restenosis lesion, number of treated vessels, number of stents and stent type, whether as a categorical variable or as a continuous variable [Model 3, OR (95% CI), *P* value: 1.786 (1.134, 2.814), 0.012 and 1.408 (1.034, 1.917), 0.030].

**Table 2:** Association of TyG with in-stent restenosis in multivariable logistic regression models.

Variables	Model 1		Model 2		Model 3	
	OR (95% CI)	*P* value	OR (95% CI)	*P* value	OR (95% CI)	*P* value
Lower TyG	Ref	–	Ref	–	Ref	–
Higher TyG^a^	1.916 (1.251, 2.933)	0.003	2.006 (1.300, 3.096)	0.002	1.786 (1.134, 2.814)	0.012
TyG^b^	1.486 (1.114, 1.982)	0.007	1.554 (1.160, 2.083)	0.003	1.408 (1.034, 1.917)	0.030

Model 1: adjusted for age and sex; Model 2: adjusted for age, sex, smoking, diabetes, hypertension, stroke and previous PCI. Model 3: adjusted for age, sex, smoking, diabetes, hypertension, stroke, previous PCI, clopidogrel, eGFR, HDL-C, restenosis lesion, number of treated vessels, number of stents and stent type. TyG, triglyceride-glucose index; PCI, percutaneous coronary intervention; eGFR, estimated glomerular filtration rate; HDL-C, high-density lipoprotein cholesterol; OR, odds ratio; CI, confidence interval.

^a^ The OR was examined regarding lower TyG as reference.

^b^ The OR was examined by per 1-unit increase of TyG.

**Figure 1:**
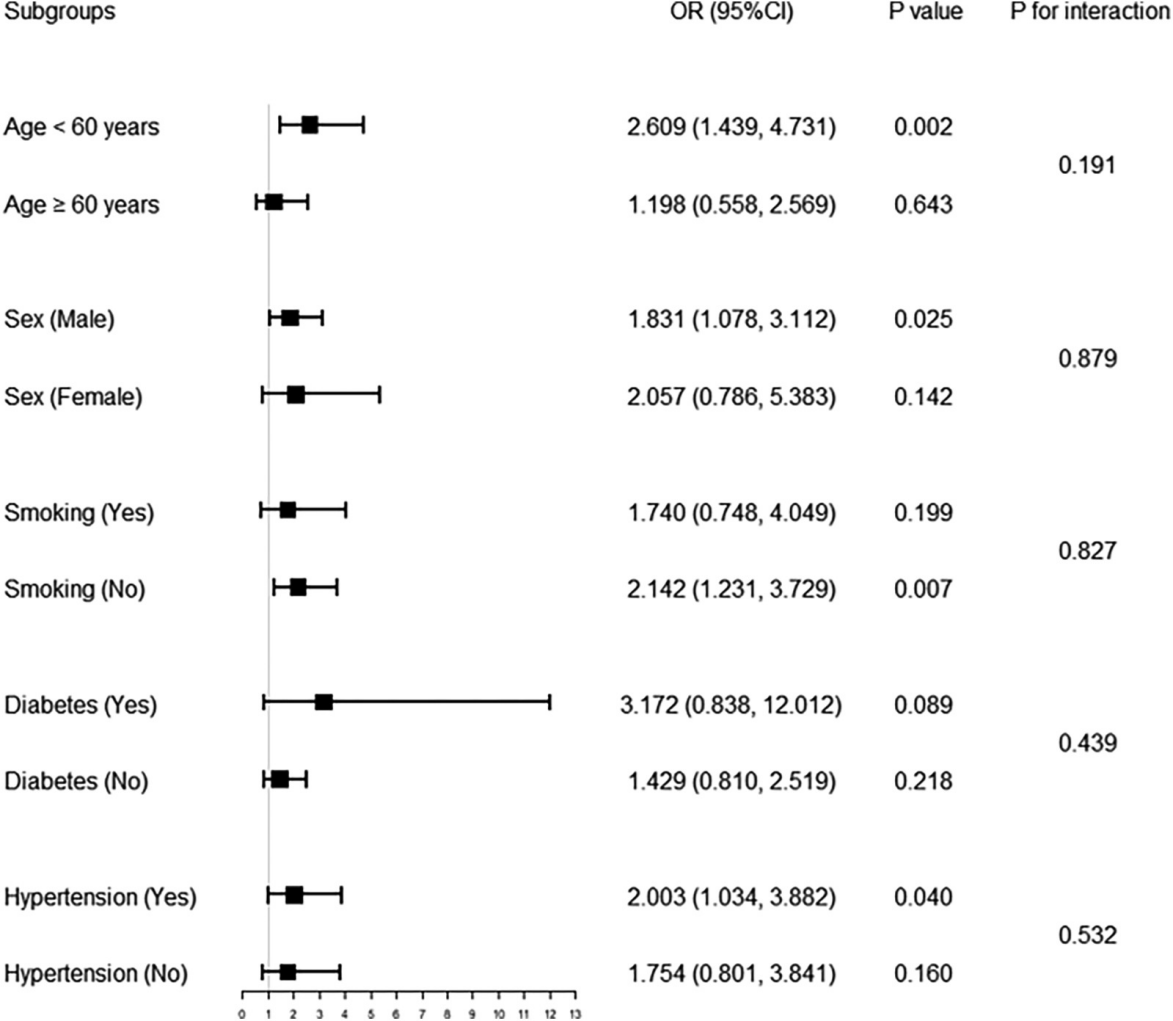
Forest plot of association of TyG with in-stent restenosis in different subgroups. Multivariable logistic regression analysis was adjusted for age, sex, smoking, diabetes, hypertension, stroke, previous PCI, clopidogrel, eGFR, HDL-C, restenosis lesion, number of treated vessels, number of stents, and stent type. TyG, triglyceride-glucose index; PCI, percutaneous coronary intervention; eGFR, estimated glomerular filtration rate; HDL-C, high-density lipoprotein cholesterol; OR, odds ratio; CI, confidence interval.

Further subgroup analyses (Figure 1) showed that in the fully adjusted model—excluding stratified variables as covariates—the association between higher TyG and increased ISR risk remained significant in the following subgroups: age <60 years, male, non-smokers and patients with hypertension [OR (95% CI), *P* value: 2.609 (1.439, 4.731), 0.002; 1.831 (1.078, 3.112), 0.025; 2.142 (1.231, 3.729), 0.007; 2.003 (1.034, 3.882), 0.040; respectively].

**Figure 2:**
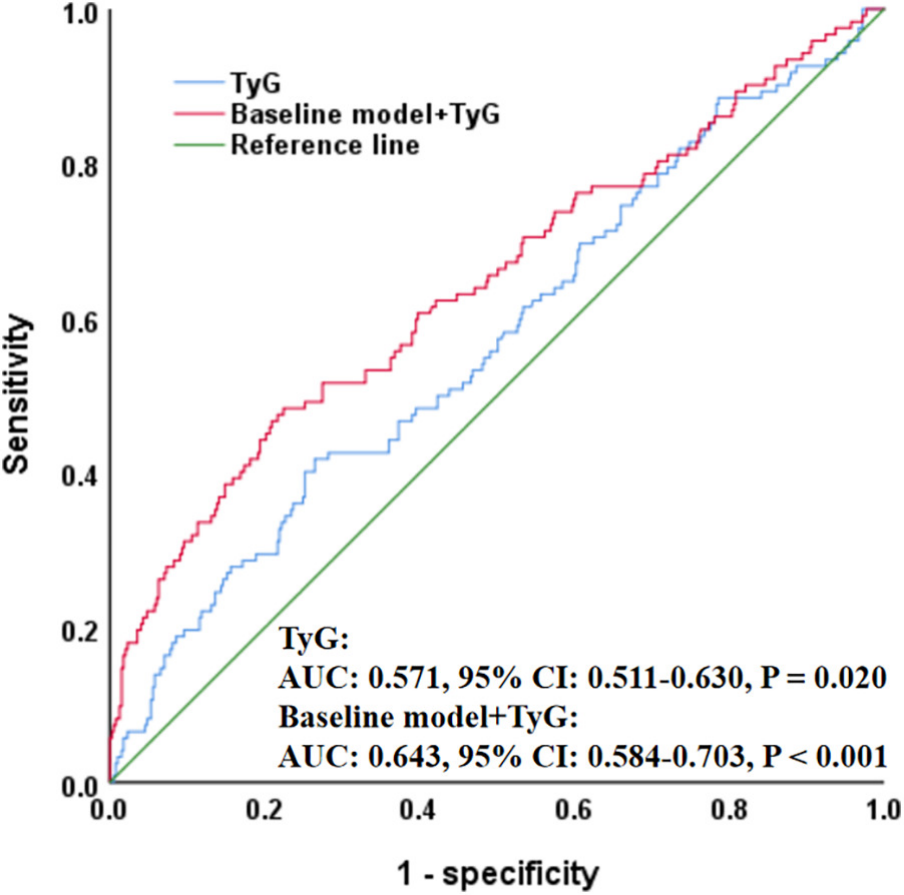
ROC curve evaluating predictive effect of TyG and baseline model for in-stent restenosis. The baseline model included age, sex, smoking, diabetes, hypertension, stroke, previous PCI, clopidogrel, eGFR, HDL-C, restenosis lesion, number of treated vessels, number of stents, and stent type. ROC, receiver operating characteristic; TyG, triglyceride-glucose index; PCI, percutaneous coronary intervention; eGFR, estimated glomerular filtration rate; HDL-C, high-density lipoprotein cholesterol; AUC, area under the curve; CI, confidence interval.

**Figure 3:**
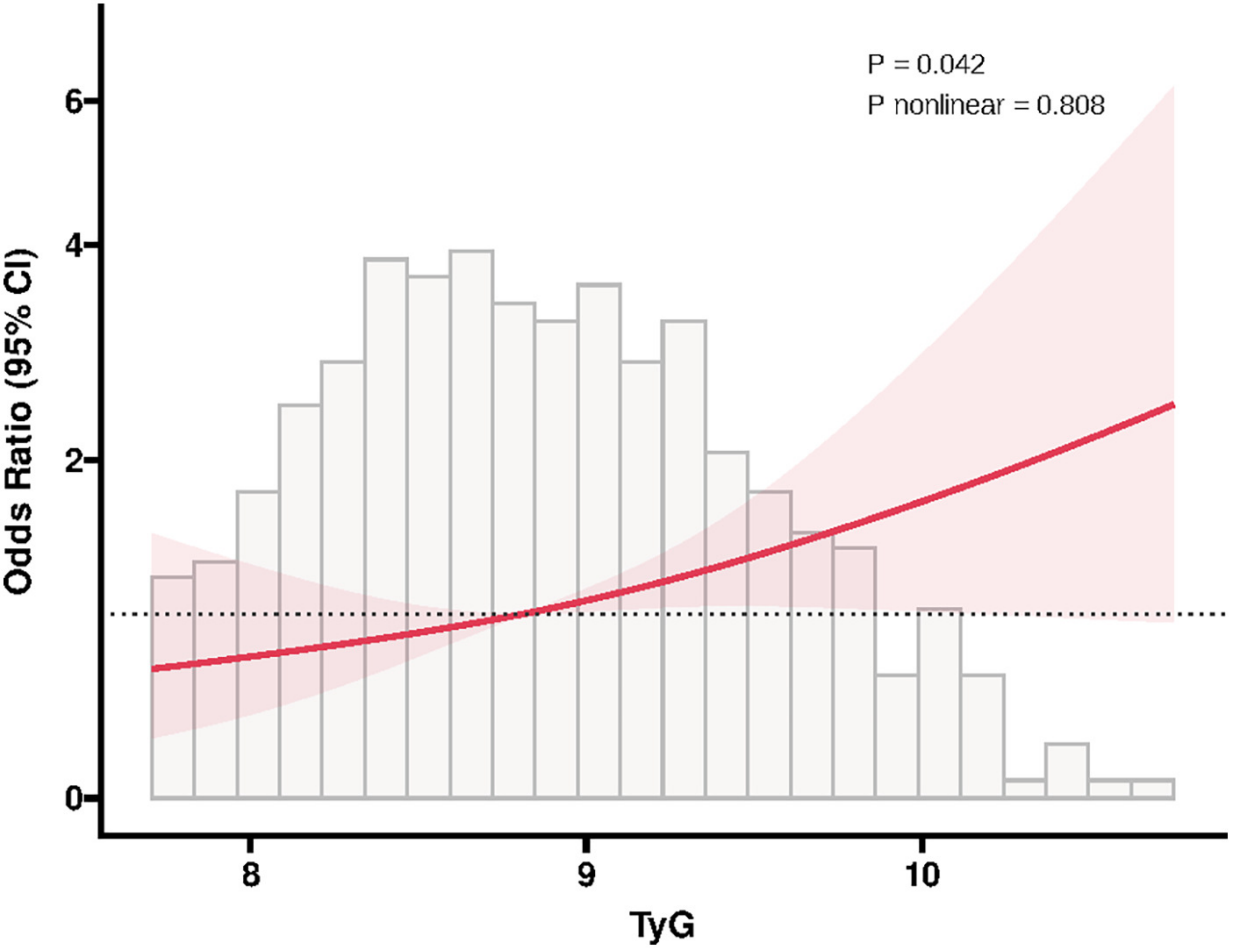
RCS plot of the association between TyG and in-stent restenosis. The RCS model was adjusted for the same covariates as in multivariable logistic regression. RCS, restricted cubic spline; TyG, triglyceride-glucose index; CI, confidence interval.

In addition, ROC analysis showed that TyG had the ability to predict ISR occurrence (AUC = 0.571, *P* = 0.020), and its addition to the baseline model (including age, sex, smoking, diabetes, hypertension, stroke, previous PCI, clopidogrel use, eGFR, HDL-C, restenosis lesion, number of treated vessels, number of stents, and stent type) significantly improved the overall predictive performance (AUC = 0.643, *P* < 0.001) (Figure 2). Moreover, RCS analysis further revealed a positive linear correlation between TyG and ISR risk (*P* = 0.042, *P* nonlinear = 0.808) (Figure 3).

## 4 Discussion

In this cohort study, we found that a higher TyG index was not only significantly associated with an increased risk of ISR in the overall population, but also remained significantly associated with the occurrence of ISR in subgroups including individuals aged <60 years, males, non-smoker, and patients with hypertension. These findings suggest that insulin resistance (IR) may play a role in the development and progression of ISR.

It is well known that diabetes contributes substantially to the burden of cardiovascular disease. Despite the widespread development and use of hypoglycemic drugs, the incidence of cardiovascular disease, as well as all-cause and cardiovascular mortality, remains significantly higher in patients with diabetes than in those without (6). In addition to the well-established microvascular and macrovascular complications of diabetes, such as neuropathy and nephropathy, its impact on ISR has garnered increasing attention (5, 22). With the advancement of coronary interventional techniques, revascularization procedures have become widespread, and the annual number of stent implantations has increased globally. While these interventions have helped salvage myocardial tissue and reduce premature death among patients with CHD, ISR remains a serious complication that can undermine the long-term benefits of PCI and increase healthcare burdens (4). Therefore, early identification and management of modifiable ISR risk factors is critical. Although the pathogenesis of ISR remains incompletely understood, diabetes has been consistently identified an independent risk factor (5, 23). For example, several studies have reported that patients with diabetes are more likely to develop ISR and experience faster lumen narrowing at the stent site (24–26). In addition, ISR in patients with diabetes more frequently occurs at the stent edge (1). However, even optimal glycemic control after PCI does not entirely eliminate ISR risk (27). Given that up to 50% of ISR cases are asymptomatic, metabolic abnormalities such as IR should be incorporated into ISR risk management (28). Several studies have indicated that residual cholesterol, high-sensitivity C-reactive protein, and uric acid may contribute to ISR pathophysiology (29–31). In this context, blood glucose, glycosylated hemoglobin and IR, which are closely related to diabetes, are inextricably linked to the risk of ISR. Although evidence on the association between these factors and ISR varies, most studies agree that higher glucose and glycosylated hemoglobin levels are independently associated with increased ISR risk (5, 32). Currently, there is no universally accepted tool for evaluating IR, and research on its association with ISR remains limited. In a small cohort of 417 CHD patients, Zhao et al. found a significant association between HOMA-IR and ISR after PCI (33). Besides, Zhu et al. used TyG as an alternative marker of IR and demonstrated a significant, independent association between elevated TyG and ISR risk in a large cohort with 2-year follow-up (34). Furthermore, Wu et al. further confirmed this link in acute coronary syndrome patients (35). In addition, Qu et al. analyzed a cohort of 830 patients who underwent carotid endarterectomy or carotid artery stenting and found that TyG was not only independently associated with the risk of restenosis after carotid revascularization, but also had predictive value for restenosis occurrence, although its predictive performance was relatively low (AUC = 0.619) (36). Similarly, Zhu et al. also reported that although TyG was significantly associated with the risk of ISR, its predictive value was limited (AUC = 0.571) (34).

Consistent with these findings, our study also demonstrated a close association between TyG and ISR, with stable correlations observed in specific subgroups such as patients aged <60 years, males, non-smokers, or those with hypertension. Interestingly, the observed elevated ISR risk in non-smokers with high TyG levels was statistically significant, while it was not significant among smokers. However, we emphasize that the *P* for interaction was 0.827, indicating no significant effect modification by smoking status. This suggests that the observed difference between non-smokers and smokers may be due to residual confounding or sample variation rather than a true interaction. Several hypotheses may explain this phenomenon: (1) Metabolic confounding: Non-smokers may have a higher burden of unmeasured metabolic abnormalities (e.g., central obesity, low physical activity, or chronic low-grade inflammation), making TyG a more sensitive indicator of IR in this group and thus more strongly associated with ISR risk; (2) Residual confounding: Due to the retrospective design of our study, we were unable to account for all potential confounding factors (such as diet, physical activity, and socioeconomic status), which may have had a greater impact on the TyG-ISR relationship in non-smokers; (3) Statistical fluctuation: The number of ISR events within each subgroup was limited, especially in smokers, which led to wider confidence intervals and lower statistical power, potentially contributing to instability in the subgroup results; (4) Selection bias: Differences in medication adherence, type of stents implanted, or follow-up management between smokers and non-smokers may have affected ISR outcomes independently of TyG levels. Therefore, further sensitivity analyses and more refined subgroup classifications (e.g., former vs. current smokers, smoking duration, smoking cessation status, etc.) would help clarify this result. However, due to the retrospective nature of our study and the lack of detailed smoking-related variables in the dataset, we were unable to perform such analyses in this study. Future prospective studies should collect more comprehensive lifestyle and metabolic data to validate this finding more thoroughly.

Additionally, although ROC analysis in our study indicated that while TyG had some predictive value for ISR occurrence, its overall predictive performance is relatively limited (AUC = 0.571). However, when TyG was added to the baseline model—which included age, sex, smoking, diabetes, hypertension, stroke, previous PCI, clopidogrel use, eGFR, HDL-C, restenosis lesion, number of treated vessels, number of stents, and stent type—the AUC significantly increased from 0.571 to 0.643, suggesting that TyG provides incremental predictive value beyond traditional clinical variables. We interpreted this result cautiously and believe it reflects the multifactorial nature of ISR pathophysiology. Therefore, to enhance its clinical utility, TyG may be better utilized as part of a composite index or integrated into existing ISR risk prediction models, thereby improving its applicability within clinical risk assessment frameworks. Nevertheless, because few studies have explored the correlation between IR and ISR, and different studies have used varied IR markers, with no comprehensive evaluation through meta-analysis or systematic review, it remains difficult to confirm the exact relationship between IR and ISR, nor can we determine the causal relationship between them. Therefore, if IR is to be incorporated into ISR risk management and treatment strategies, more multicenter, large-sample, prospective clinical studies are needed to further clarify this association.

Although this study provides valuable findings, our understanding of the pathophysiological mechanisms linking IR and ISR remains incomplete. Diabetes and IR are known to exert various deleterious vascular effects that significantly contribute to the development of cardiovascular diseases, including ISR. These mechanisms include: (1) Endothelial dysfunction: Hyperglycemia and hyperinsulinemia reduce nitric oxide (NO) bioavailability, impairing vasodilation, increasing endothelial permeability, and promoting leukocyte adhesion and vascular inflammation (37, 38). (2) Chronic inflammation and oxidative stress: Diabetes and IR induce low-grade systemic inflammation and elevated reactive oxygen species (ROS), which stimulate vascular smooth muscle cell (VSMC) proliferation, migration, and extracellular matrix deposition—key processes in neointimal hyperplasia and restenosis (39, 40). (3) Pro-thrombotic and anti-fibrinolytic state: IR is associated with increased procoagulant factors and suppressed fibrinolytic activity, facilitating thrombus formation at the stent site and enhancing the risk of ISR (41). (4) Dyslipidemia and accelerated atherosclerosis: IR often accompanies elevated TG and low HDL-C, which promote lipid accumulation, plaque instability, and contribute to the progression of neoatherosclerosis within the stent (39). (5) Abnormal vascular remodeling: IR-related signaling disturbances, including phosphatidylinositol 3-kinase (PI3K)/protein kinase B (Akt) and mitogen-activated protein kinase (MAPK) pathways, impair endothelial repair, delay re-endothelialization, and disrupt vascular healing, exacerbating restenosis risk (42). Given these mechanisms, the TyG index—as a simple and accessible surrogate marker of IR—may capture key metabolic and inflammatory perturbations contributing to ISR development. Our findings, which demonstrated a significant association between higher TyG levels and increased ISR risk, particularly in certain subgroups, likely reflect the cumulative impact of these underlying vascular pathologies. To deepen mechanistic insights, future studies should incorporate more detailed metabolic and inflammatory profiling, and consider experimental models or prospective cohorts to further elucidate the causal pathways linking IR and ISR.

Regardless of these strengths, our study has some limitations. First, as an observational study, it cannot establish a causal relationship between TyG and ISR. Second, although TyG is a validated surrogate marker of IR, it is not the gold standard, and we did not compare it with other IR markers such as fasting insulin or HOMA-IR, which may weaken the interpretation of TyG as a superior predictor of ISR risk. This limitation was primarily due to the retrospective nature of our study and the absence of fasting insulin data in the existing dataset. Third, the relatively small sample size may limit the generalizability of our findings. Fourth, the finding that non-smokers exhibited a higher risk of ISR despite a higher TyG level is intriguing and paradoxical. This could be due to other confounding factors, such as metabolic disorders, inflammation, or IR, that might play a more prominent role in the non-smoker population. Particularly, in non-smokers, TyG could serve as a stronger predictor of ISR risk due to underlying metabolic issues. This result warrants further investigation and discussion. In this regard, recent work by Presch et al. provides valuable insights (43). Their study found that smoking at baseline was associated with increased risks of all-cause death, cardiovascular death, myocardial infarction, and stent thrombosis, while also showing a lower risk of repeat revascularization in smokers. These findings indicate a complex relationship between smoking and vascular outcomes. Their study suggests that while smoking may increase long-term risks, smoking cessation could lead to different outcomes. Similarly, in our study, the higher ISR risk in non-smokers with elevated TyG levels may reflect underlying metabolic disturbances rather than the direct effects of smoking. Moreover, we were unable to perform a subgroup analysis of former smokers (those who quit smoking post-stent implantation) due to the limitations of our retrospective study design. Future studies should consider exploring the effects of smoking cessation on ISR risk through a prospective design and analyzing the relationship between smoking history and metabolic markers such as TyG. Fifth, ROC analysis revealed a modest AUC of 0.571 for TyG alone, suggesting limited standalone predictive utility. However, even in the combined model, the overall predictive performance remained suboptimal, suggesting that TyG alone or in combination with clinical features may not fully capture the complex pathophysiology of ISR. To improve clinical applicability, TyG should ideally be compared with composite indices, such as TyG to waist-to-height ratio (TyG-WHtR), TyG to waist circumference (TyG-WC), TyG to a body shape index (TyG-ABSI), or C-reactive protein to TyG (CRP-TyG), which may better capture the multifactorial pathophysiology of ISR. Unfortunately, this is a retrospective study based on a previously established cohort, and CRP and WC were not recorded for most patients at the time of data collection. Therefore, we were unable to calculate or include these composite indices in the current analysis. Additionally, although BMI is a commonly used composite index, we found a significant number of missing values for BMI, and no significant association was observed between TyG-BMI and ISR. Therefore, we did not include these composite indices in the analysis. In future prospective studies, we plan to systematically collect additional key metabolic and inflammatory parameters, including CRP, and WC, to construct and validate more comprehensive composite indices. This will allow for more accurate modeling of ISR risk and improved understanding of the interplay between IR and vascular outcomes. Sixth, although the median follow-up time in this study was 29.8 months, we did not collect specific ISR event occurrence times or individual patient follow-up durations. Therefore, survival analysis methods, such as Kaplan–Meier curves or Cox proportional hazards models, were not applicable in this study due to the lack of detailed event time data. Additionally, we did not perform serial TyG measurements, so we were unable to analyze TyG trajectories, such as rising vs. stable levels, and their relationship with ISR risk. In addition, due to the limitations of clinical studies, we may have uncontrollably missed some risk factors for ISR, such as dietary habits and coronary plaque burden, which were not available in our dataset and may have influenced the results. Finally, the study by Zhu et al. found that the significant association between TyG and ISR was present only in non-diabetic patients with acute coronary syndrome, while no such association was observed in diabetic patients (34). Additionally, Zhao et al. demonstrated that TyG was significantly associated with the short-term risk of restenosis after carotid artery stenting, regardless of diabetes status (44). However, in our study, the association between TyG and ISR did not reach statistical significance in either the diabetic or non-diabetic subgroups. This outcome may be influenced by several factors. On one hand, diabetic patients represented a relatively small proportion of the overall study population, while non-diabetic patients, despite being more numerous, had fewer ISR events. These factors may have led to insufficient statistical power in both subgroups. On the other hand, in the diabetic subgroup, variations in glucose-lowering therapies (such as oral medications vs. insulin) and differences in treatment adherence could have interfered with the independent predictive ability of TyG for ISR. In the non-diabetic group, unmeasured confounding factors such as dietary habits, physical activity, and lipid metabolism may also have weakened the observed associations. due to the retrospective design, important confounders such as diet, physical activity, and glycemic management were not available and could have influenced the associations. Furthermore, the pathophysiology of ISR is highly complex, and a single metabolic indicator may not be sufficient to fully capture its multifactorial nature. Importantly, the *P* for interaction between diabetes status and TyG was 0.439, indicating no significant effect modification by diabetes, but this does not preclude the possibility of unmeasured confounding. It is also important to note that this study was based on a retrospective dataset, and we were unable to increase the sample size beyond the existing population. Therefore, we recommend that future studies with larger and more balanced sample sizes, particularly in diabetic subgroups, be conducted to better elucidate the potential modifying role of diabetes. Moreover, prospective designs with detailed data on antidiabetic therapies, treatment adherence, and other lifestyle factors may provide deeper insight into the complex interaction between TyG and ISR risk in diabetic vs. non-diabetic populations.

## 5 Conclusions

In this real-world study involving hospitalized patients, our findings demonstrated a significant association between the TyG and ISR following PCI in patients with CHD. This not only reinforces the potential association between IR and ISR but also provides additional evidence supporting the detrimental impact of IR on cardiovascular events. And these findings also provide valuable insights and a theoretical foundation for future evidence-based approaches and precision medicine strategies.

## Data Availability

Publicly available datasets were analyzed in this study. This data can be found here: Dryad repository, https://doi.org/10.5061/dryad.13d31.
